# Supplementary material for: Single planar photonic chip with tailored angular transmission for multiple-order analog spatial differentiator
Source: Nat Commun. 2022 Dec 26;13:7944. doi: 10.1038/s41467-022-35588-5 (PMC9792592; doi:10.1038/s41467-022-35588-5)
Supplement: Supplementary file 1 — Supplementary Information [file 41467_2022_35588_MOESM1_ESM.pdf]

Supplementary Information for

Single planar photonic chip with tailored angular transmission for multiple-order analog spatial differentiator

Yang Liu[1], Mingchuan Huang[1], Qiankun Chen[1], and Douguo Zhang[1,2]†

[1]Advanced Laser Technology Laboratory of Anhui Province, Department of Optics and Optical Engineering, University of Science and Technology of China, Hefei, Anhui, 230026, China

[2] Hefei National Laboratory, University of Science and Technology of China, Hefei 230088, China

†Correspondence and requests for materials should be addressed to: dgzhang@ustc.edu.cn (Douguo Zhang)

Section 1: General theoretical analysis of analog spatial differentiation

When the electric field $E_{in}(x,y)$ in Cartesian coordinate system carrying the object information is incident on the photonic chip, the output transmitted electric field $E_{out}(x,y)$ can be derived based on the spatial filtering capability of the chip. Using a Fourier expansion, the incident and transmitted electric fields can be written as the sum of a large number of plane waves as follows:

$$E_{in}(x,y) = \iint A(k_x,k_y)\exp(ik_x x + ik_y y)dk_x dk_y \ . \tag{S1.1}$$

$$E_{out}(x,y) = \iint t(k_x,k_y)A(k_x,k_y)\exp(ik_x x + ik_y y)dk_x dk_y \ . \tag{S1.2}$$

where $A(k_x,k_y)$ represents the amplitude of the electric field, and $t(k_x,k_y)$ is the optical transfer function (OTF).

To perform the first-order differential operation along the x-direction, we must design a photonic chip in which the transmitted field has the profile $E_{out} \propto \frac{\partial E_{in}}{\partial x}$, and the optical transfer function must satisfy the following condition:

$$t(k_x,k_y) \propto k_x \tag{S1.3}$$

In the same way, to achieve higher-order one-dimensional differential operations, $t(k_x,k_y)$ should have the following forms:

$$t(k_x,k_y) \propto k_x^2 \tag{S1.4}$$

$$t(k_x,k_y) \propto k_x^3 \tag{S1.5}$$

Specifically, to perform the two-dimensional second-order differential operation, the photonic chip should play the role of a Laplacian operator with a transmitted profile of $E_{out} \propto \nabla^2 E_{in}$, where ∇^2 is the Laplacian operator given by $\partial_x^2 + \partial_y^2$. In this case, the optical transfer function must satisfy the following condition:

$$t(k_x,k_y) \propto k_x^2 + k_y^2 = k_r^2 \tag{S1.6}$$

In the polar coordinate system, the output transmitted electric field is re-written as:

$$E_{out}(k_r,\varphi)=\iint t(k_r,\varphi)A(k_r,\varphi)\exp[ik_r r\cos(\alpha-\varphi)]k_r dk_r d\varphi \tag{S1.7}$$

where $k_r=\sqrt{k_x^2+k_y^2}$ is the wave vector along the in-plane radial direction, $\alpha=\arctan\left(\frac{x}{y}\right)$ is the angle between polar axis r and y-axis in real space, $\varphi=\arctan\left(\frac{k_x}{k_y}\right)$ is the angle between polar axis k_r and k_y-axis in fourier space as shown in Fig. S1a.

Similarly, to achieve multi-order radial differential operations ($E_{out}\propto\frac{\partial^n E_{in}}{\partial r^n}$) which enable two-dimensional processing, the optical transfer function should satisfy the forms of $t(k_r,\varphi)\propto k_r^1,k_r^2,k_r^3,k_r^4,\cdots\cdots$. It is worth noting that for a pure phase object, the output field after the first-order differential operation can be written as:

$$E_{out}=\frac{\partial E_{in}}{\partial x}=ie^{i\phi}\frac{\partial\phi}{\partial x} \tag{S1.8}$$

$$I=\left|\frac{\partial\phi}{\partial x}\right|^2 \tag{S1.9}$$

Therefore, the detected intensity of the phase object after implementation of the first-order differentiation operation is the square of the modulus of its phase gradient, which carries the object's phase information.

Section 2: Theoretical analysis on the optical transfer function of the photonic chip

The photonic chips used in this work are composed of alternating Si_3N_4 and SiO_2 layers, which have different refractive indices and thicknesses. The guided resonance inside this multilayer structure affects the transmission and reflection behavior of the electric field, and this behavior is analyzed using temporal coupled-mode theory[1]. Two processes occur when the light beam passes through this photonic chip. The first process is direct transmission, where part of the incident energy

is transmitted through the chip directly. The second process is indirect transmission, in which the remaining energy from the incident beam induces guided resonances. The interference between these two processes determines the transmission spectrum of this photonic chip, which can be written as follows:

$$t = t_d + f \frac{\gamma(\mathbf{k})}{i(\omega_0 - \omega(\mathbf{k})) + \gamma(\mathbf{k})} \tag{S2.1}$$

$$f = -(t_d \pm r_d) \tag{S2.2}$$

where t_d is the direct transmission coefficient, f is the complex amplitude of the resonant mode, ω_0 is the frequency of the incident electric field, $\omega(\mathbf{k})$ is the center frequency, and γ is the radiative linewidth of the resonance.

The angle of incidence can be written as $\theta = \arcsin(\frac{|\mathbf{k}_r|}{|\mathbf{k}_0|})$, and the frequency at incidence can be expressed as $\omega_0 = \omega(\mathbf{k} = \mathbf{k_0})$. By performing a Taylor expansion, the transmission t around $\mathbf{k} = \mathbf{k_0}$ is obtained as follows:

$$t(\omega_0, \mathbf{k}) = \mp r_d - (t_d \pm r_d) \frac{i}{\gamma(\mathbf{k}_0)} \delta\omega(\mathbf{k}) \tag{S2.3}$$

where the first term represents the direct transmission process, and the second term determines the line type of the transmission curve. If the condition that $\delta\omega(\mathbf{k}) \propto |\mathbf{k_0}|^2$ is met, then the transmission t will have a quadratic linear form. Similarly, the transmission t will have a linear form if the condition that $\delta\omega(\mathbf{k}) \propto |\mathbf{k_0}|$ is satisfied.

To determine the desired line type, we simulated the transmission spectrum of the photonic chip using the transfer matrix method (TMM)[2, 3]. Assuming that the two materials used in the chip are arranged periodically along the z-direction, the relative permittivity and thickness of the i-th material layer are ε_i and d_i, respectively. Benefiting from the continuity of the electric field at the

interface, we can then correlate the electric field $\begin{pmatrix} a_0 \\ b_0 \end{pmatrix}$ at the location of incidence with the electric field $\begin{pmatrix} a_n \\ 0 \end{pmatrix}$ at the output location; here, a_0 and b_0 represent the incident and reflected electric field amplitudes in the first layer, respectively, and a_n is the amplitude of the output electric field from the last (n-th) layer.

$$\begin{aligned}\begin{pmatrix} a_0 \\ b_0 \end{pmatrix} &= \mathbf{D_0^{-1}} \prod_{i=1}^{n-1} \mathbf{D_i P_i^{-1} D_i^{-1} D_n} \begin{pmatrix} a_n \\ 0 \end{pmatrix} \\ &= \mathbf{M} \begin{pmatrix} a_n \\ 0 \end{pmatrix} \\ &= \begin{pmatrix} \mathbf{M_{11}} & \mathbf{M_{12}} \\ \mathbf{M_{21}} & \mathbf{M_{22}} \end{pmatrix} \begin{pmatrix} a_n \\ 0 \end{pmatrix}\end{aligned} \tag{S2.4}$$

where $\mathbf{D_i} = \begin{pmatrix} 1 & 1 \\ p_i & -p_i \end{pmatrix}$, $\mathbf{P_i} = \begin{pmatrix} e^{ip_i d_i} & 0 \\ 0 & e^{-ip_i d_i} \end{pmatrix}$, $P_i = \sqrt{\varepsilon_i k_0^2 - \beta^2}$, and β is the propagation constant of the electromagnetic field. $\mathbf{M}$ in the equation above is the transfer matrix of this process.

Because the transmission coefficient is the ratio of the transmitted field amplitude to the incident field amplitude, it can be obtained as:

$$t = \frac{a_n}{a_0} = \frac{1}{\mathbf{M_{11}}} \tag{S2.5}$$

The transmission band diagram was obtained using the TMM (Fig. 1b).A transmission curve that satisfies the quadratic type ($t = t_0 + t_2 k_r^2$) can be obtained at the frequency of 467 THz (λ = 643 nm) (see Fig. S1b). The existing directly transmitted (DC) component t_0 will become the background, which will damage the spatial differentiation effect, and it is thus necessary to suppress the DC component in this case. Based on the calculated transmissivity, the angle-dependent output electric field distribution can be determined from the incident electric field (Fig. 1c and 1d consistent with the experimental BFP images). In the simulations, the refractive index values used for the Si_3N_4 and

SiO_2 layers (n = 2.53 and 1.46, respectively) were consistent with the experimental values measured via ellipsometry. The thicknesses of the Si_3N_4 and SiO_2 layers were set at 56 nm and 80 nm, respectively. A total of 20 pairs of Si_3N_4 + SiO_2 periodic layers were placed on the substrate glass (refractive index of 1.515, thickness of 0.17 mm). The thicknesses of each of the layers and the total number of layers are consistent with the SEM image of the fabricated photonic chip.

Section 3: Deflection of polarization orientation of the light transmitted through the chip

In this photonic chip, the transmission t_s (for the s-polarized light) is different to the transmission t_p (for the p-polarized light). When a linearly polarized light field E_0 (with polarization orientation along the y-direction) is incident on the chip at the azimuthal angle φ, the electric field can be decomposed into two orthogonal components with the s- and p-polarizations, and the electric field passing through the chip can then be written as:

$$E_s = t_s E_0 \sin(\varphi) \tag{S3.1}$$

$$E_p = t_p E_0 \cos(\varphi) \tag{S3.2}$$

$$E_{out} = \sqrt{E_s^2 + E_p^2} \tag{S3.3}$$

The polarization direction (φ') of the output beam is deflected from that of the incident beam (see Fig. S2), as illustrated by Eq. S3.4

$$\varphi' = \arctan(\frac{E_s}{E_p}) \neq \varphi \tag{S3.4}$$

In the case of nearly normal incidence, the difference between t_s and t_p is very small, and thus the polarization direction after transmission through the chip is deflected only slightly ($\varphi' \approx \varphi$). To suppress the DC component of the transmitted light, an analyzer with an orientation that lies perpendicular to the polarization direction of the incident light is inserted into the system after the

collection objective. This analyzer can erase the DC component while simultaneously maintaining the quadratic line type at the wavelength of 643 nm, thus allowing the second-order spatial differentiation operation with a high signal-to-noise ratio to be achieved.

In the case of oblique incidence, the difference between t_s and t_p becomes larger, and the polarization direction is obviously deflected after transmission. The analyzer in this case not only erases the DC component, but also extends the range of quadratic line type to larger numerical aperture by modifying the transmission curve (Fig. S1c and S1d). It is worth noting that the polarization direction is not deflected along the horizontal and vertical directions, but is largely deflected along other azimuth angles. The analyzer therefore modulates the transmittance spectrum tangentially. As a result, the first-order spatial differential operation can then be implemented along the horizontal and vertical directions (Fig. S1e).

Section 4: A precise analysis on the origin of the multi-order differentiations

The above is the qualitative description, a more precise explanation is presented here. Eqs. S1.1 and S1.2 can be rewritten as the vector field forms:

$$\mathbf{E}_{\mathbf{in}}(x,y)=\mathbf{e}_{\mathbf{in}}\iint A(k_x,k_y)\exp(ik_xx+ik_yy)dk_xdk_y \ . \tag{S4.1}$$

$$\mathbf{E}_{\mathbf{out}}(x,y)=\mathbf{e}_{\mathbf{out}}\iint t(k_x,k_y)A(k_x,k_y)\exp(ik_xx+ik_yy)dk_xdk_y \ . \tag{S4.2}$$

where $\mathbf{e_{in}}$ and $\mathbf{e_{out}}$ represent the polarization states of input and output fields in real space, which are controlled by the two polarizers used in the experiment (Fig. 2c and Fig. 3a)

Considering Eqs. S4.1 and S4.2, the optical transfer function relates the input and output fields:

$$t(k_x,k_y)=\mathbf{e}_{\mathbf{out}}^{\dagger}\mathbf{M}^{-1}\mathbf{T}(k_x,k_y)\mathbf{M}\mathbf{e}_{\mathbf{in}} \tag{S4.3}$$

where $\mathbf{T}\left(k_x,k_y\right)=\begin{bmatrix} t_s\left(k_x,k_y\right) & 0 \\ 0 & t_p\left(k_x,k_y\right)\end{bmatrix}$ is the matrix of transmission coefficients in the basis of s, p basis, and $\mathbf{M}$ is the transformation matrix converting from the x, y basis to the s, p basis:

$$\mathbf{M}=\begin{bmatrix} -\cos\varphi & \sin\varphi \\ \sin\varphi(1+\frac{\theta^2}{2}) & \cos\varphi(1+\frac{\theta^2}{2})\end{bmatrix} \tag{S4.4}$$

Combing Eqs. S4.3 and S4.4, the optic transfer function is obtained[4]:

$$\begin{aligned} t(k_x,k_y) &= \mathbf{e}_{\mathbf{out}}^{\mathbf{x}*}\mathbf{e}_{\mathbf{in}}^{\mathbf{x}}(t_s\cos^2\varphi+t_p\sin^2\varphi) \\ &+\mathbf{e}_{\mathbf{out}}^{\mathbf{x}*}\mathbf{e}_{\mathbf{in}}^{\mathbf{y}}\left(\frac{\sin 2\varphi}{2}(t_p-t_s)\right) \\ &+\mathbf{e}_{\mathbf{out}}^{\mathbf{y}*}\mathbf{e}_{\mathbf{in}}^{\mathbf{x}}\left(\frac{\sin 2\varphi}{2}(t_p-t_s)\right) \\ &+\mathbf{e}_{\mathbf{out}}^{\mathbf{y}*}\mathbf{e}_{\mathbf{in}}^{\mathbf{y}}(t_s\sin^2\varphi+t_p\cos^2\varphi) \end{aligned} \tag{S4.5}$$

In the state where the input and output light are orthogonal linearly polarized ($\mathbf{e}_{\mathbf{in}}=\begin{bmatrix}0\\1\end{bmatrix}$ and $\mathbf{e}_{\mathbf{out}}=\begin{bmatrix}1\\0\end{bmatrix}$, determined by the polarizer 1 and polarizer 2 in Fig. 2c and Fig. 3a) ,

$$t(k_x,k_y)=\frac{\sin 2\varphi}{2}\left(t_p-t_s\right) \tag{S4.6}$$

Consider the horizontal symmetry of the structure, the Taylor expansions of the transmission coefficients $t_{s(p)}$ have only even terms:

$$t_{s(p)}=t_0+C_{s2(p2)}\theta^2+C_{s4(p4)}\theta^4+\cdots\cdots+C_{s2n(p2n)}\theta^{2n},\ n\in N^+ \tag{S4.7}$$

where t_0 is the directly transmitted component at normal incidence, $\theta=\arcsin\left(\frac{k_r}{k_0}\right)\approx\frac{k_r}{k_0}$ and $C_{s2n(p2n)}$ are the coefficients of even terms.

Thus, the difference in transmission coefficients (t_p-t_s) can be written in the form of a polynomial:

$$t_p-t_s=\left(C_{p2}-C_{s2}\right)\theta^2+\left(C_{p4}-C_{s4}\right)\theta^4+\cdots\cdots+\left(C_{p2n}-C_{s2n}\right)\theta^{2n} \tag{S4.8}$$

When the wavelength of incident light is adjusted to 643 nm, the difference ($t_p - t_s$) is in the quadratic form and the other expansion terms are small (see Fig. S3a and S3d, based on the simulation results by TMM):

$$t_p - t_s = \left(C_{p2} - C_{s2}\right)\theta^2 \tag{S4.9}$$

Combing Eqs. S4.5, S4.6 and S4.9, the optic transfer function at λ = 643 nm can be obtained:

$$t(k_x, k_y) = \frac{\sin 2\varphi}{2}\left(C_{p2} - C_{s2}\right)\theta^2 \tag{S4.10}$$

When the azimuth angle φ is fixed as a constant, the optical transfer function has the following form:

$$t = a \bullet k_r^2,\ a = \frac{\left(C_{p2} - C_{s2}\right)}{2k_0^2} \bullet \sin 2\varphi \tag{S4.11}$$

When the vertical wave vector k_y is fixed as a constant, the form of the optical transfer function can be written as:

$$t = b \bullet k_x,\ b = \frac{\left(C_{p2} - C_{s2}\right)}{k_0^2} \bullet k_y \tag{S4.12}$$

A conclusion can be made from Eqs. S4.11 and S4.12: At the wavelength of 643 nm, the optic transfer function has a quadratic form along the radial direction that corresponds to the second-order spatial differential operation. In the meantime, it has a linear form along the direction of $\mathbf{k_x}$, which corresponds to the first-order spatial differential operation.

When the wavelength of incident light is adjusted to 638 nm, the difference ($t_p - t_s$) is in the quartic form and the other expansion terms are small (see Fig. S3b and S3e, based on the simulation results by TMM):

$$t_p - t_s = \left(C_{p4} - C_{s4}\right)\theta^4 \tag{S4.13}$$

Similarly, the optic transfer function at λ = 638 nm is obtained:

$$t(k_x,k_y)=\left(C_{p4}-C_{s4}\right)\frac{\sin 2\varphi}{2}\theta^4=\left(C_{p4}-C_{s4}\right)\bullet\left[\left(\frac{k_x}{k_0}\right)^3\frac{k_y}{k_0}+\frac{k_x}{k_0}\left(\frac{k_y}{k_0}\right)^3\right] \tag{S4.14}$$

When the azimuth angle φ is fixed as a constant, the optical transfer function has the following form:

$$t=c\bullet k_r^4,\ c=\frac{\left(C_{p4}-C_{s4}\right)}{2k_0^4}\bullet\sin 2\varphi \tag{S4.15}$$

Considering the term $\frac{k_y}{k_0}\in\left[-0.25,0.25\right]$ in this optical system, the term $\frac{k_x}{k_0}\left(\frac{k_y}{k_0}\right)^3$ is two orders of magnitude smaller than the term $\left(\frac{k_x}{k_0}\right)^3\frac{k_y}{k_0}$ when k_y is a constant. Then, Eq. S4.14 can be written as:

$$t=d\bullet k_x^3,\ d=\frac{\left(C_{p4}-C_{s4}\right)}{k_0^4}\bullet k_y \tag{S4.16}$$

Then, a conclusion can be made from Eqs. S4.15 and S4.16: At the wavelength of 638 nm, the optic transfer function has a quartic form along the radial direction that corresponds to the fourth-order spatial differential operation. It also has a cubic form along the direction of $\mathbf{k_x}$, which corresponds to the third-order spatial differential operation.

By using the same principle, the difference in transmission coefficients (t_p-t_s) satisfied $t_p-t_s=\left(C_{p6}-C_{s6}\right)\theta^6$ (all other terms are small in quantity except for the sixth power term) could be find at λ = 635 nm (see Fig. S3c and S3f). Then, the optic transfer function can be written in the form:

$$t(k_x,k_y)=\left(C_{p6}-C_{s6}\right)\frac{\sin 2\varphi}{2}\theta^6=\begin{cases} e\bullet k_r^6,\ e=\frac{\left(C_{p6}-C_{s6}\right)}{2k_0^6}\bullet\sin 2\varphi \\ f\bullet k_x^5,\ f=\frac{\left(C_{p6}-C_{s6}\right)}{k_0^6}\bullet k_y \end{cases} \tag{S4.17}$$

Therefore, the fifth- and the sixth-order differential operations can be implemented. In the same

way, higher order differential operations become possible that is very helpful in Taylor-expanding the data to get finer details.

Section 5: Resolution of multi-order differentiation

An issue worthy of attention is "What is the resolution of such multi-order differentiations?". Inspired by the description of figure of merit (FOM)[5] in the imaging process, we proposed a method to define the resolution of multi-order differentiations.

A one-dimensional rectangular pulse wave function $E_{in}^{x}(x) = p(x) = \Pi\left(x/2x_0\right)$ (Fig. S6a) is used to replace the field with information of USAF Resolution Test Target, which also can be regarded as the sum of a large number of plane waves:

$$E_{in}^{x}(x) = \int_{-\infty}^{\infty} P(k_x) e^{-ik_x x} dk_x \tag{S5.1}$$

where $P(k_x)$ is the spatial spectra of the incident field obtained using the spatial Fourier transform:

$$\begin{aligned} P(k_x) &= \frac{1}{2\pi}\int_{-\infty}^{\infty} p(x) e^{ik_x x} dx \\ &= \frac{\sin k_x x_0}{\pi k_x} \\ &= \frac{x_0}{\pi} sinc(\frac{x_0}{\pi} k_x) \end{aligned} \tag{S5.2}$$

The output field E_{out}^{x} modulated by OTF is calculated as:

$$E_{out}^{x}(x) = \int_{-k_{\max}}^{k_{\max}} t(k_x) P(k_x) e^{-ik_x x} dk_x \tag{S5.3}$$

where the OTF is written as $t(k_x) = ik_x^n$ where n represents the order of multi-order differentiations and k_{max} is the maximum transverse wave vector acceptable by the system, which is determined by the aperture of the collection system.

The normalized output fields $|E_{out}^{x}|^2$ around the right edge of the rectangle input wave are plotted in Fig. S6b-S6e where the number of edges corresponding to the order of the multi-order differential and the diffraction fringes are displayed. The definition of resolution can refer to the

Rayleigh criterion in which the edges are only detectable until the maximum point of one edge signal coincides with the first zero of another edge signals. Therefore, we define the distance between the peak of the peripheral edge and the position of the first zero value as Δx_n, the distance between center and boundary of the edge is L_n, and the resolution of multi-order differentiation is obtained as $R = (2L_n - \Delta x_n)/\lambda$ (normalized to the incident wavelength λ_0). Finally, the theoretical resolution of the first-, second-, third- and forth- order differential are 1.10 (0.71 μm), 2.35 (1.51 μm), 3.52 (2.25 μm) and 4.64 (3.00 μm),respectively. Moreover, the analysis results of resolution in Fig. S6b-S6e are consistent with that in Fig. 3i and 3h and in Fig. 4d and 4c.

Section 6: Method of wavelength customization

In the previous section, we discussed the differential case at specific wavelengths, and then a question will be raised: Can we customize the operation wavelength to obtain a chip that works at the desired wavelength?

In order to answer this question, we sorted out the process of chip design. Firstly, two materials with known refractive index were selected as the basic periodic structures of one-dimensional photonic crystals (the proposed planar photonic chip). As the widely used materials, Si_3N_4 and SiO_2 were selected due to the low cost and mature processing technology for thin films deposition. Secondly, a total number of 40 layers of this chip is designed to realize a large band gap depth (Fig. 1b and Fig. S8c), so that the form of the OTF changes rapidly with the wavelength to achieve the conversion of multi-order differentiations. Thirdly, the layer thicknesses of the two dielectric layers need to be determined. By adjusting the thicknesses of the two dielectric layers, the photonic band structure will move along the direction of increasing or decreasing incident light frequency. In this

way, we can tune the working frequency (or wavelength), at which the spatial differentiations can be realized. As a result, the operation wavelength of the photonic chip can be customized (Fig. S7).

References for SI reference citations

1. Fan SH, Joannopoulos JD. Analysis of guided resonances in photonic crystal slabs. *Physical Review B* 2002, **65**(23).

2. Yeh P, Yariv A, Hong CS. Electromagnetic propagation in periodic stratified media. I. General theory. *Journal of the Optical Society of America* 1977, **67**(4)**:** 423-438.

3. Anemogiannis E, Glytsis EN, Gaylord TK. Determination of guided and leaky modes in lossless and lossy planar multilayer optical waveguides: Reflection pole method and wavevector density method. *J Lightwave Technol* 1999, **17**(5)**:** 929-941.

4. Long OY, Guo C, Wang H, Fan S. Isotropic topological second-order spatial differentiator operating in transmission mode. *Optics Letters* 2021, **46**(13)**:** 3247-3250.

5. Karimi P, Khavasi A, Khaleghi SSM. Fundamental limit for gain and resolution in analog optical edge detection. *Opt Express* 2020, **28**(2)**:** 898-911.

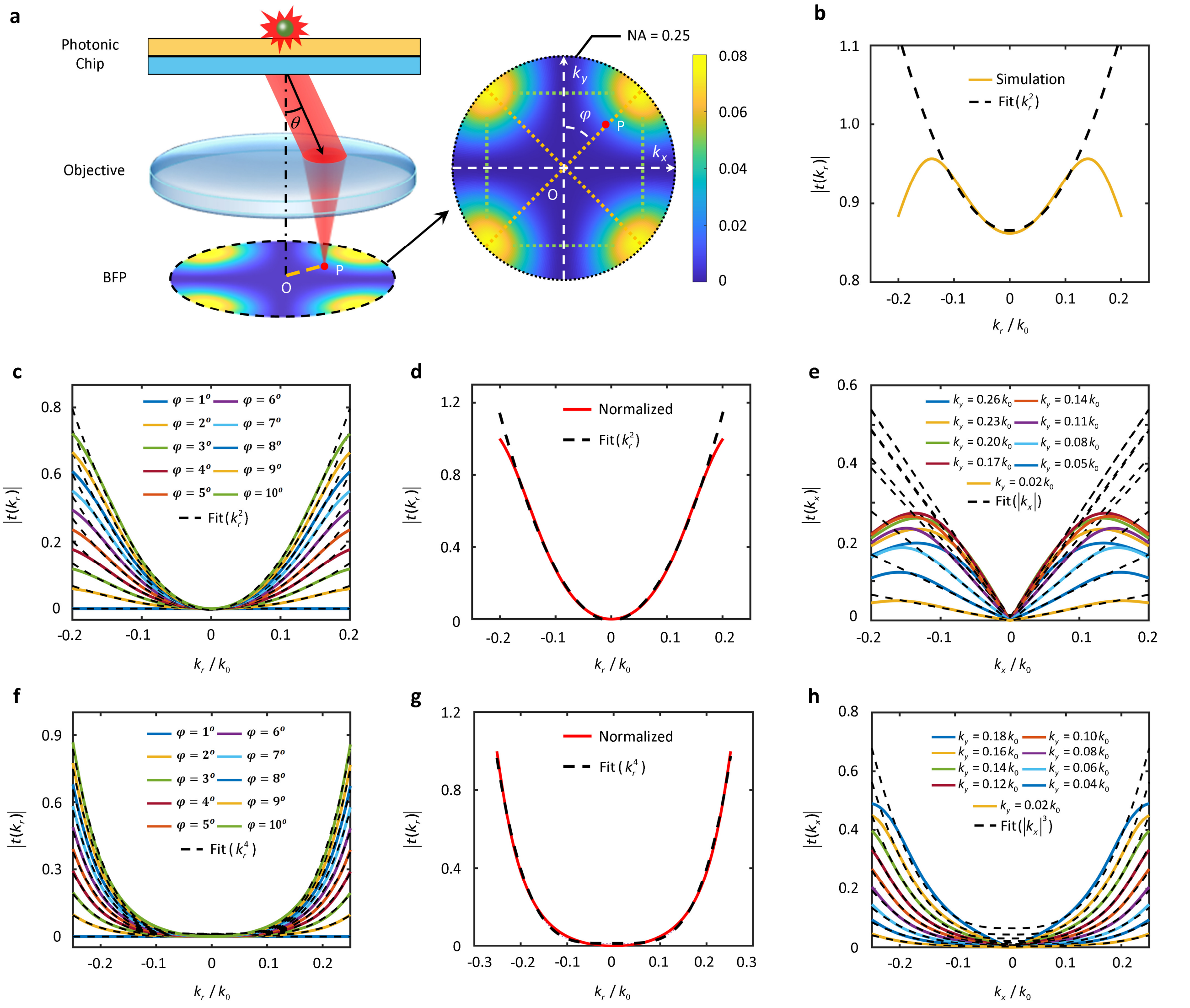

Figure S1. Calculated angle-dependent transmittance characteristics of the planar photonic chip. **a** Simulated angle-dependent transmission (also known as the transmission image in the momentum domain, corresponding to the experimentally transmitted BFP image, where the NA is within 0.25) at a wavelength of 643 nm. The green (orange) dotted lines represent the directions of incidence for the first-order (second-order) spatial differentiation. The coordinate system in the momentum domain is established using k_x and k_y, and the azimuth angle between the direction of the second-order derivative and the k_y-axis is defined as φ. Each point on (**a**), e.g., point P**,** represents one direction of incidence defined by the radial angle θ and the azimuthal angle φ, and the color is an encoded

representation of the normalized transmittance. **b** Transmission curves calculated without orthogonal polarization state along the radial direction (φ=45°) at λ = 643 nm, and the corresponding fitting curves are given by $|t| = ak_r^2$. **c**, **f** Transmission curves calculated in orthogonal polarization state along the directions marked using the yellow lines in (**a**) at (**c**) λ = 643 nm and (**f**) λ = 638 nm, where the azimuthal angle φ varies from $1°$ to $10°$ and the corresponding fitting curves are given by $|t| = ak_r^2$ and $|t| = ck_r^4$. **d**, **g** Normalized transmission curves at (**d**) λ = 643 nm and (**g**) λ = 638 nm along the directions marked using the yellow lines with various φ ranging from $10°$ to $80°$. **e**, **h** Transmission curves at (**e**) λ = 643 nm and (**h**) λ = 638 nm along the directions marked using the green lines in (**a**) with k_y varying from $0.02k_0$ to $0.26k_0$ (or from $0.02k_0$ to $0.18k_0$), and corresponding linear fitting curves given by $|t| = b|k_x|$ and $|t| = d|k_x|^3$.

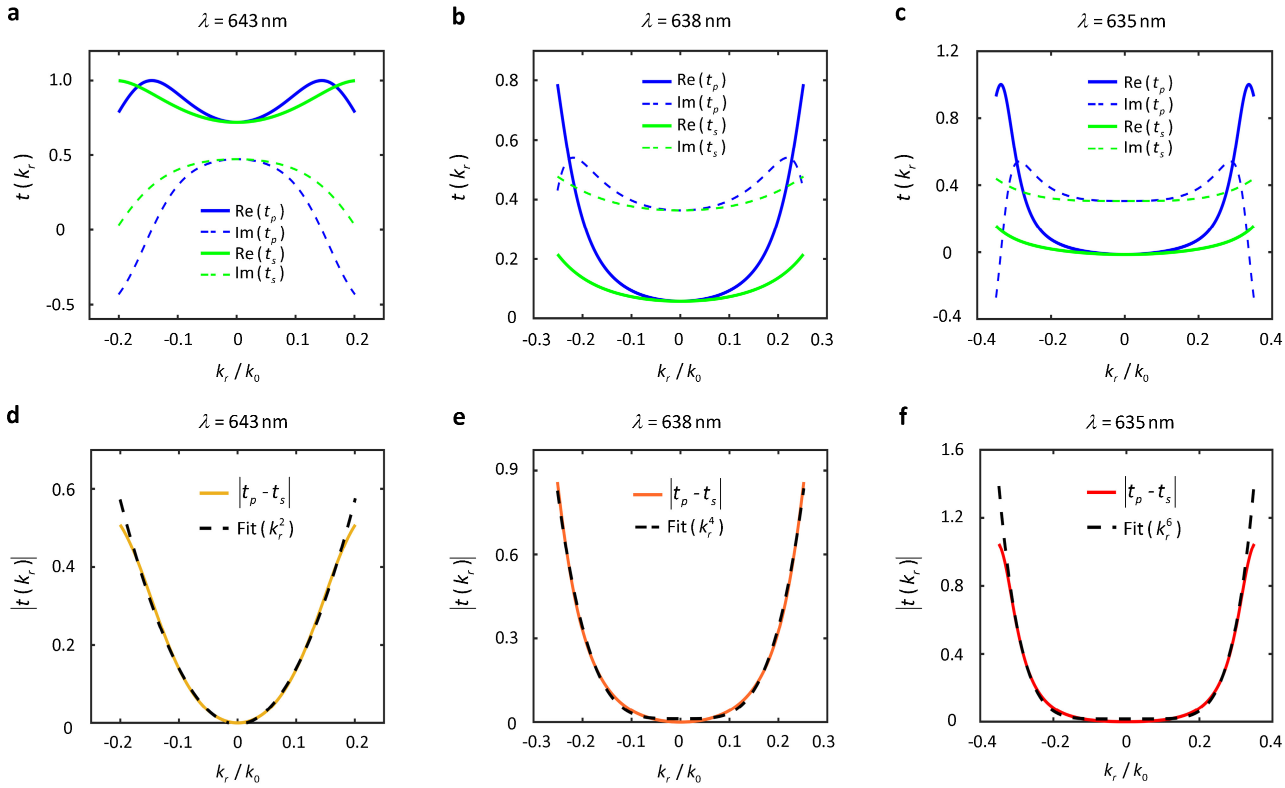

Figure S2. Transmission curves along different directions. **a**-**c** Profiles of transmissions for the s- and p-polarizations extracted from Fig. 1b at (**a**) λ = 643 nm, (**b**) λ = 638 nm and (**c**) λ = 635 nm. **d**-**f** The differences in transmission coefficients $|t_p - t_s|$ of the electric field corresponding to the quadratic, quartic and sixth line type, respectively.

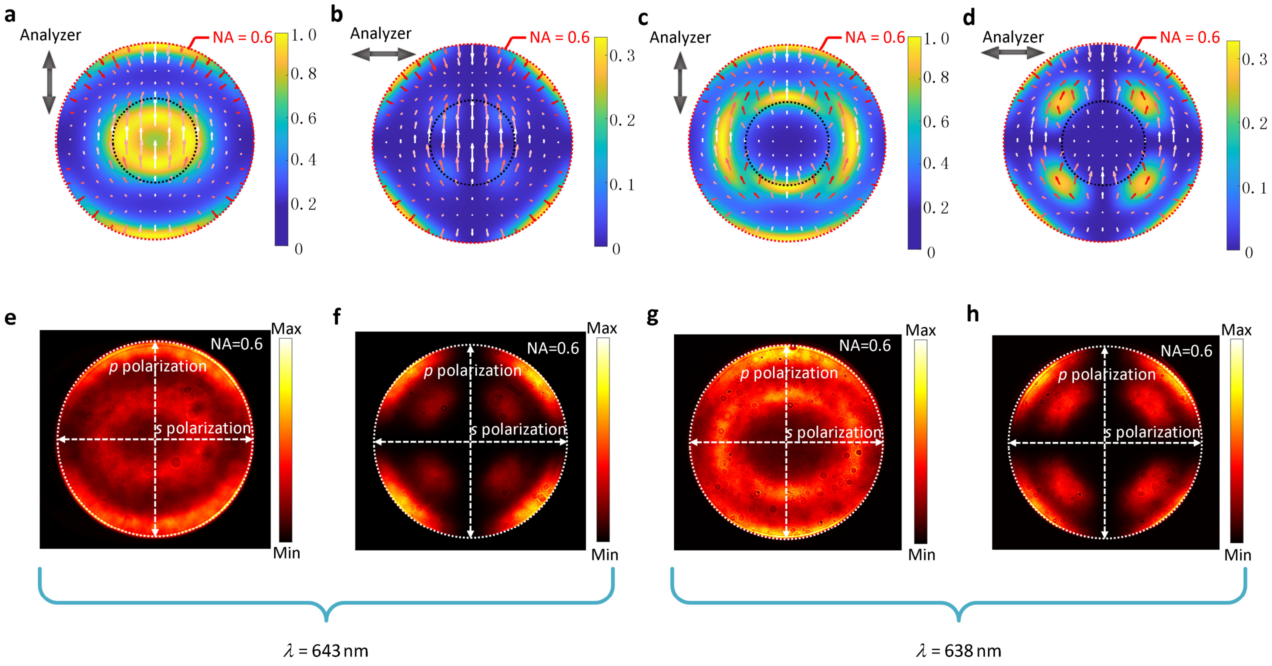

Figure S3. Comparison between simulated and measured BFP images. **a**, **b** Simulated BFP images at λ = 643 nm when the orientation of the analyzer (polarizer 2) was (**a**) parallel or (**b**) perpendicular to the orientation of polarizer 1 shown in Fig. 2c. **c**, **d** Simulated BFP images at λ = 638 nm when the orientation of the analyzer (polarizer 2) was (**c**) parallel or (**d**) perpendicular to the orientation of polarizer 1.The positions of $\mathrm{NA}=0.6$ in the spectrums are marked by red dotted circles and the positions of $\mathrm{NA}=0.25$ are marked by black dotted circles. **e**-**h** Experimentally measured BFP images corresponding to (**a**)-(**d**) that were obtained using the experimental setup shown in Fig. 2c.

$\varphi(^{\circ})$	1	2	3	4	5	6	7	8	9	10
Coefficient *a*	0.2544	0.5085	0.7619	1.0144	1.2657	1.5155	1.7634	2.0091	2.2524	2.4930

Table S1. Fitting coefficients *a* used in Fig. S1c.

k_y/k_0	0.02	0.05	0.08	0.11	0.14	0.17	0.20	0.23	0.26
Coefficient *b*	0.2757	0.7081	1.1670	1.6293	2.0175	2.2267	2.2083	2.0040	1.6988

Table S2. Fitting coefficients *b* used in Fig. S1e.

$\varphi(^{\circ})$	1	2	3	4	5	6	7	8	9	10
Coefficient *c*	2.3833	4.7637	7.1383	9.5042	11.8585	14.19842	16.5210	18.8234	21.1030	23.3568

Table S3. Fitting coefficients *c* used in Fig. S1f.

k_y/k_0	0.02	0.04	0.06	0.08	0.10	0.12	0.14	0.16	0.18
Coefficient *d*	2.5401	5.1537	8.0703	11.5780	15.6698	20.6180	26.6613	33.1133	39.1457

Table S4. Fitting coefficients *d* used in Fig. S1h.

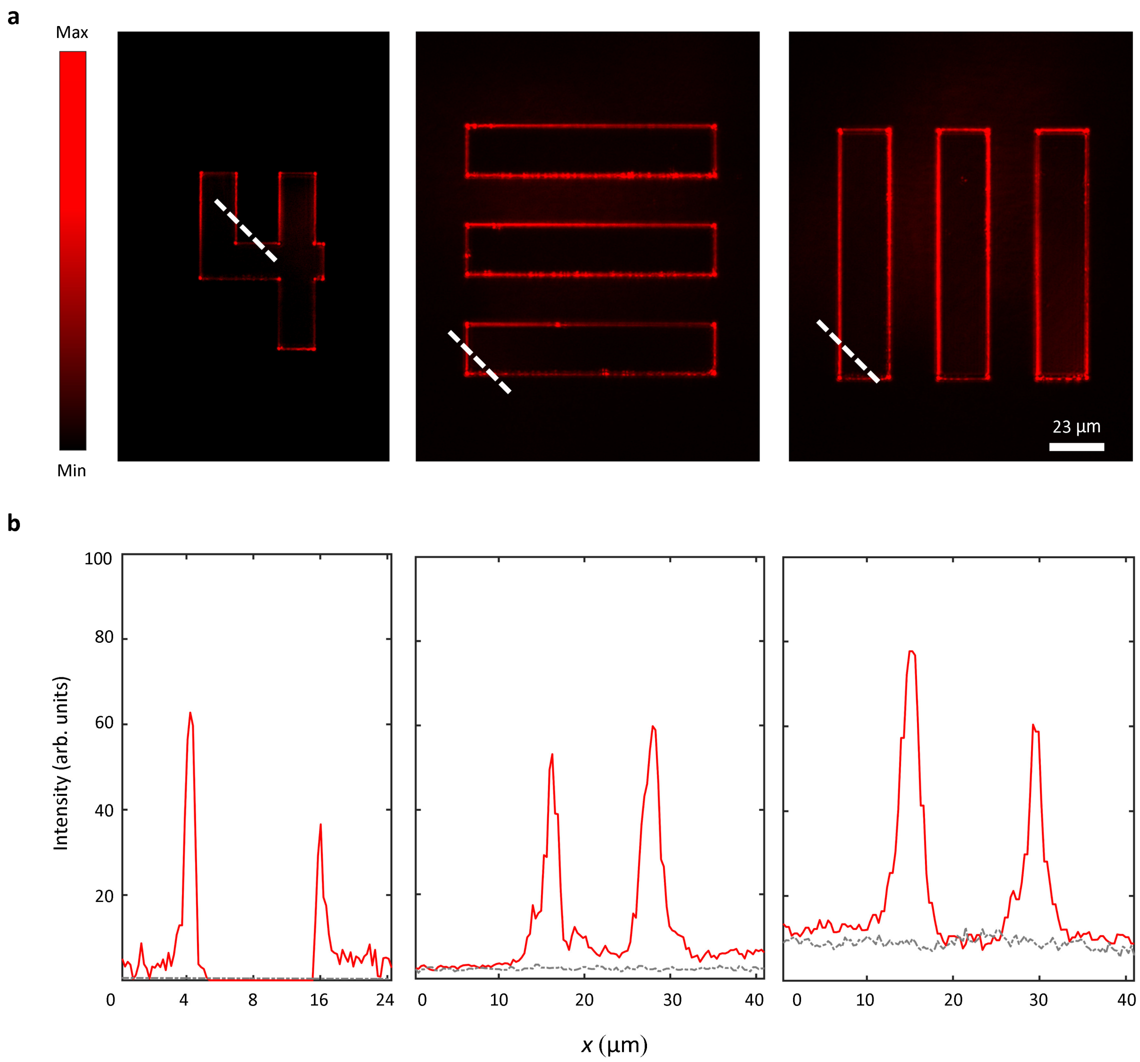

Figure S4. 2D first-order spatial differentiation. **a** Results of imaging of the 1951 USAF target with a line width of 23 µm using first-order differentiation processing in both the horizontal and vertical directions. The differentiation direction is oriented along an oblique direction. **b** Intensity profiles measured along the white dashed lines shown in (**a**). The grey dashed lines represent the background intensity. The full name of the abbreviation “arb. units” is “arbitrary units”.

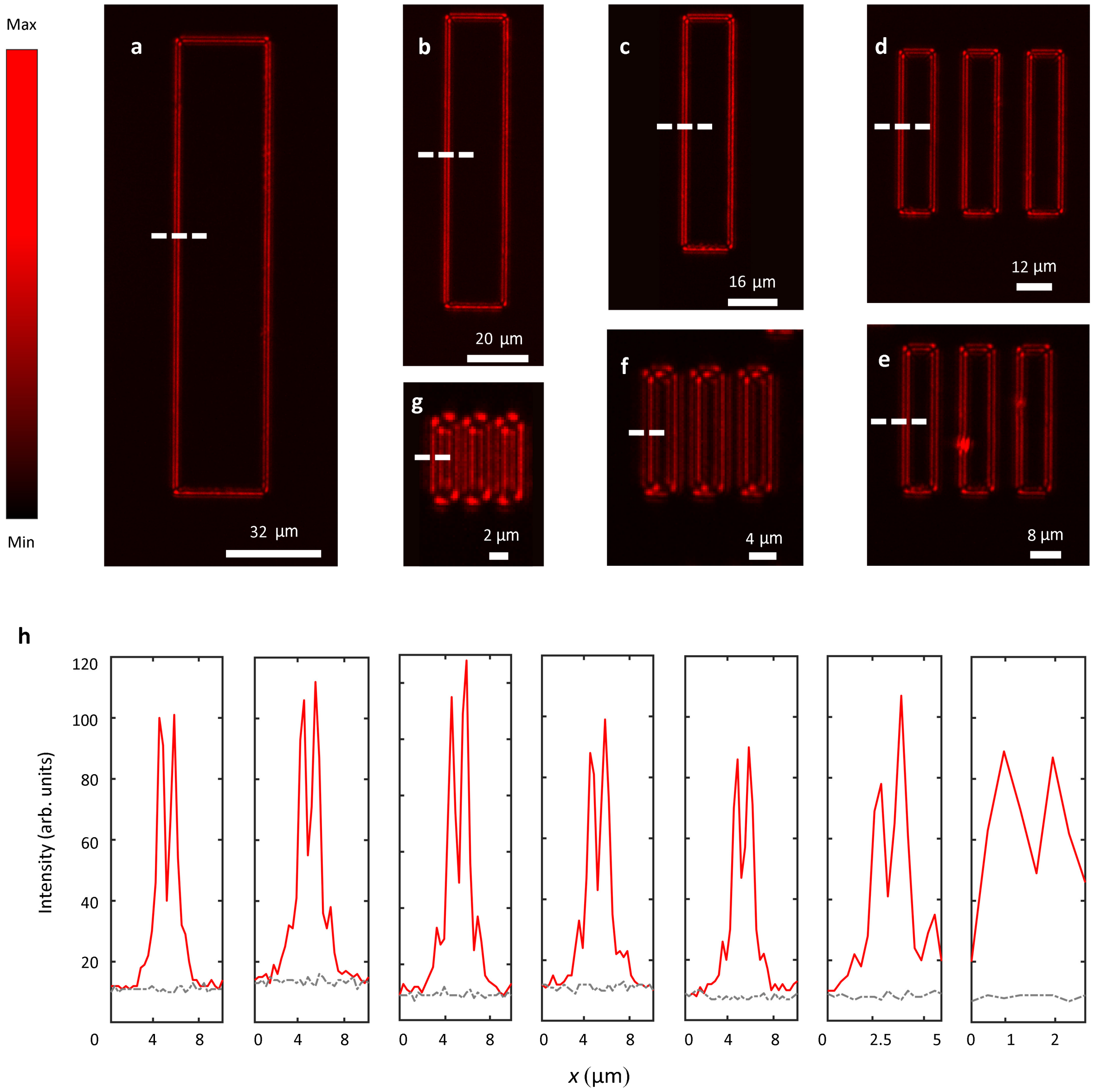

Figure S5. Differentiator resolution characterization. **a**–**g** Results of imaging of the 1951 USAF target with line widths ranging from 32 μm to 2 μm using the second-order spatial differentiation operation. **h** Intensity profiles measured along the white dashed lines marked in (**a**)–(**g**). The grey dashed lines represent the background intensity.

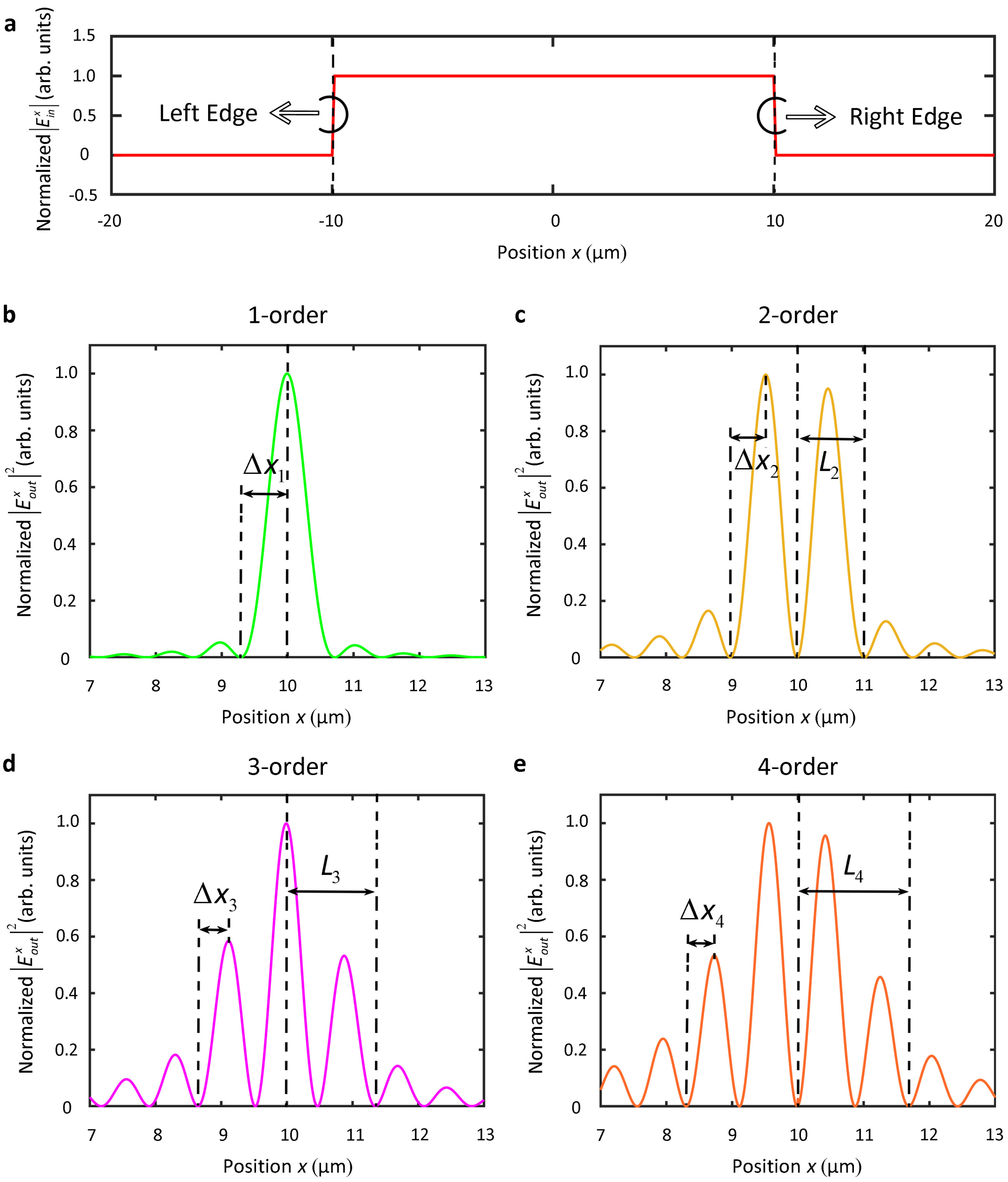

Figure S6. Analysis on the resolution of the multi-order spatial differentiations based on Rayleigh criterion. **a** Normalized rectangle incident field $|E_{in}^{x}|$ with "Left Edge" and "Right Edge" marked. **b**-**e** Normalized output field $|E_{out}^{x}|^2$ modulated by (**b**) the first-, (**c**) the second-, (**d**) the third- and (**e**) the forth-order differentiation around the "Right Edge" of the input field. Δx_n represents the distance between the peak of the peripheral edge and the position of the first zero value, and L_n represents the distance between center and boundary of the edge, where n is the order of the spatial differentiations.

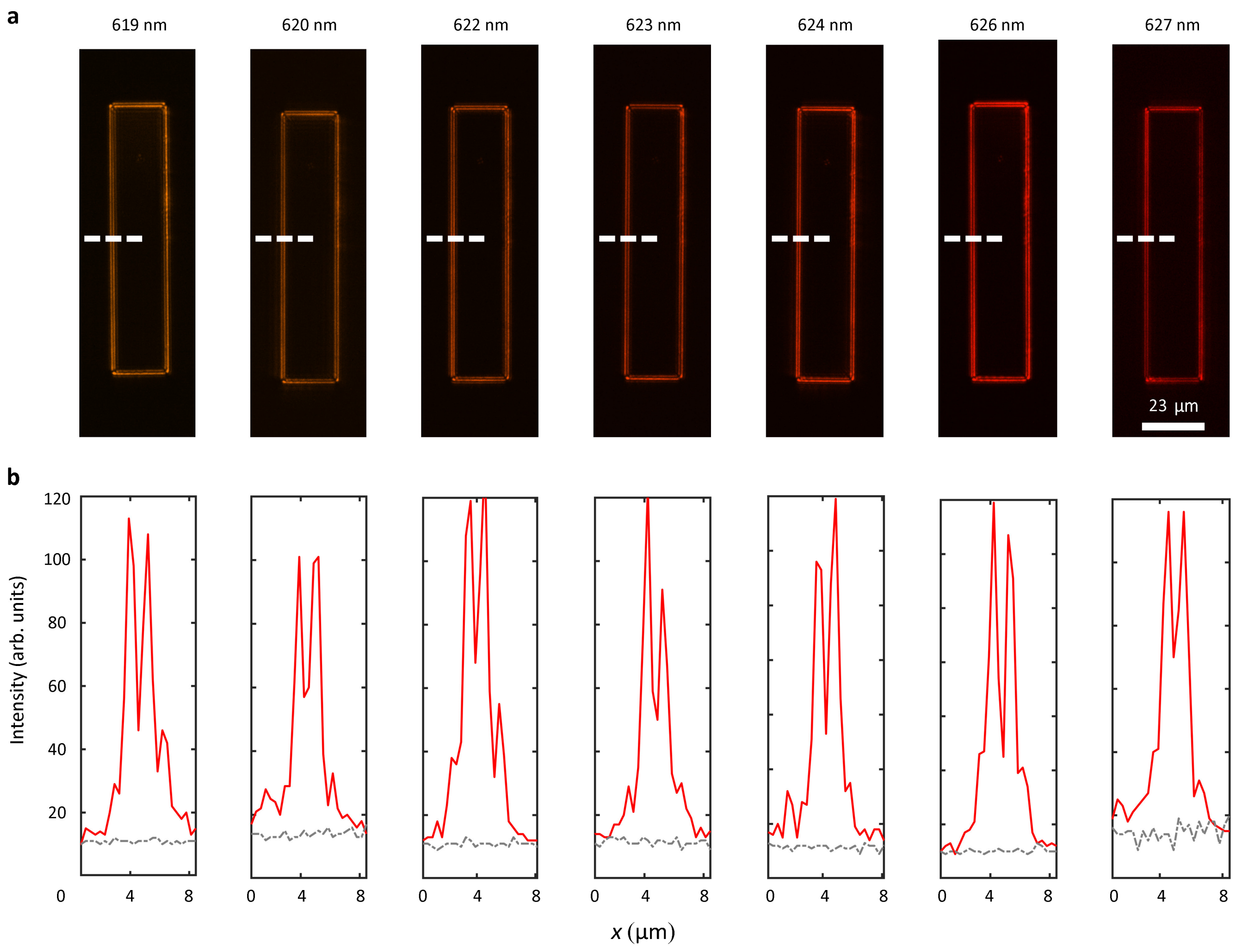

Figure S7. Implementation of second-order differentiation using different wavelengths. **a** The operating wavelength was changed from 619 nm to 627 nm with 1 nm increments by changing the thicknesses of the two materials in the photonic chips. The line width of the patterns is 23 µm. **b** Intensity profiles measured along the white dashed lines marked in (**a**). The grey dashed lines represent the background intensity.

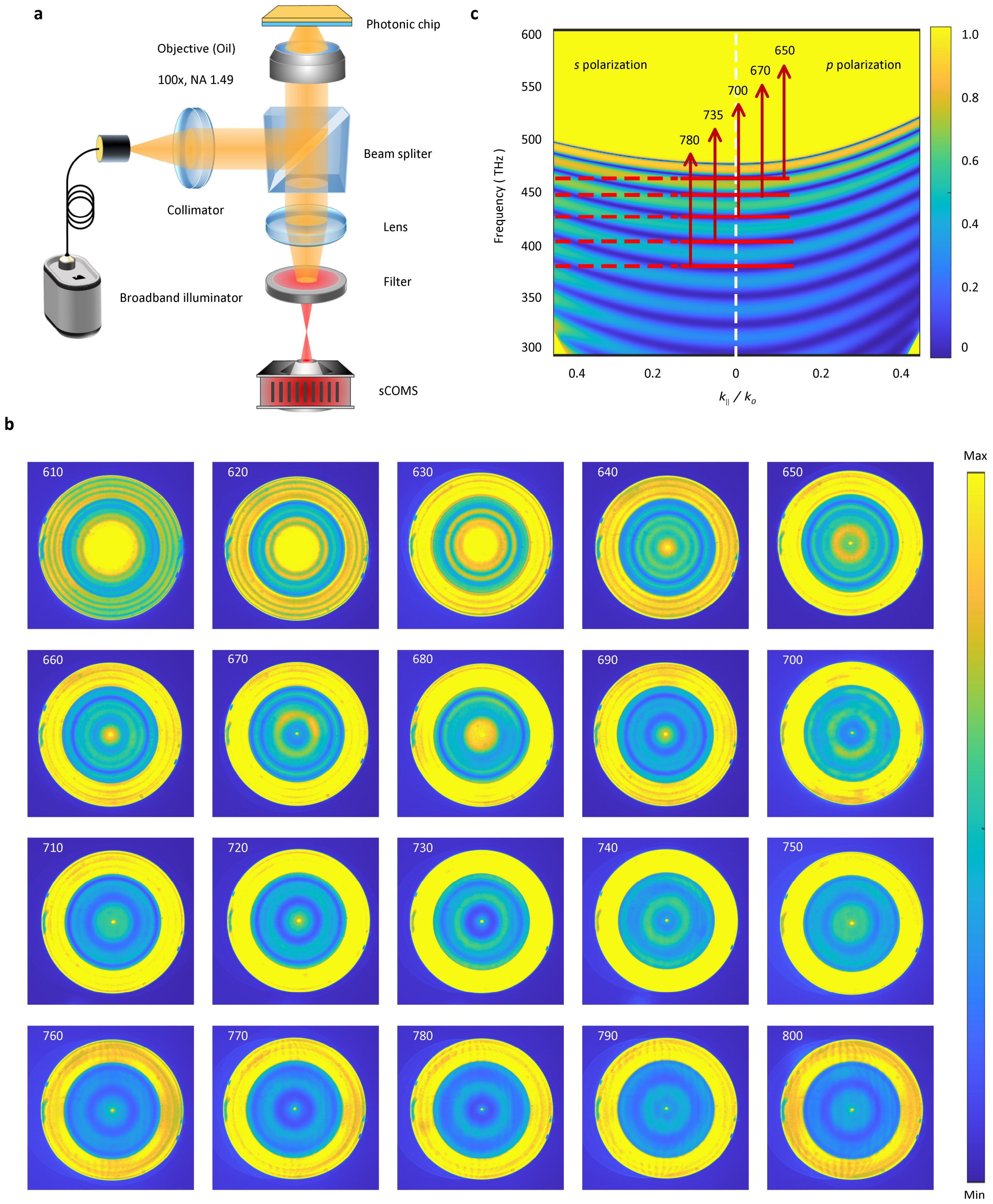

Figure S8. Reflection BFP imaging of the planar photonic chip. **a** Schematic of the reflection BFP imaging system, in which an oil-immersed objective (CFI Apochromat TIRF, 100×/NA = 1.49; Nikon) was used to measure the angular distribution of the reflected light fully. The center wavelengths of

the band-pass filters ranged from 610 to 800 nm (20 filters were used in total), with a full width at half maximum (FWHM) of 10 ± 2 nm. **b** Reflection BFP images of the fabricated chip at different incident wavelengths, where the dark centers represent low reflectivity areas. **c** Reflectance spectra calculated using the multilayer fabrication parameters. The wavelengths at which the BFPs exhibited low reflectivity near normal incidence were marked in (**c**), indicating that the fabrication results show good agreement with the pre-calculated values.
