# Supplementary material for: Single planar photonic chip with tailored angular transmission for multiple-order analog spatial differentiator
Source: Nat Commun. 2022 Dec 26;13:7944. doi: 10.1038/s41467-022-35588-5 (PMC9792592; doi:10.1038/s41467-022-35588-5)
Supplement: Supplementary file 3 — Description of Additional Supplementary Files [file 41467_2022_35588_MOESM3_ESM.pdf]

Description of Additional Supplementary Files

Supplementary Video 1: The demonstration of the second-order spatial differentiation. When the analyzer is inserted into the optical path, the optical image will be differentiated in the second-order. The orientation of the analyzer is perpendicular to that of the incident beam’s polarization.

Supplementary Video 2: The demonstration of the first-order spatial differentiation. When the analyzer is inserted into the optical path, the optical image will be differentiated in the first-order. The orientation of the analyzer is perpendicular to that of the incident beam’s polarization.

Supplementary Video 3: The demonstration of the fourth-order spatial differentiation. The optical configuration is the same as that used in the second-order spatial differentiation. When the incident wavelength is changed from 643 nm to 638 nm, the fourth-order spatial differentiation appears.

Supplementary Video 4: The demonstration of the third-order spatial differentiation. The optical configuration is the same as that used in the first-order spatial differentiation. When the incident wavelength is changed from 643 nm to 638 nm, the third-order spatial differentiation appears.
